# Effects of an enteral nutrient-rich therapy with omega-3 fatty acids in patients with unresectable or recurrent biliary tract cancer or pancreatic cancer during chemotherapy: a case–control study

**DOI:** 10.1007/s12032-021-01625-4

**Published:** 2022-04-28

**Authors:** Kyohei Abe, Tadashi Uwagawa, Ryoga Hamura, Yoshihiro Shirai, Jungo Yasuda, Kenei Furukawa, Hironori Shiozaki, Shinji Onda, Takeshi Gocho, Toru Ikegami

**Affiliations:** Department of Surgery, The Jikei University School of Medicine, 3-25-8, Nishi-Shinbashi, Minato-ku, Tokyo, 105-8461 Japan

**Keywords:** Hepatobiliary and pancreatic cancer, Omega-3 fatty acids, Cancer cachexia

## Abstract

To evaluate omega-3 fatty acid-rich enteral nutrient effects in patients with unresectable or recurrent biliary tract or pancreatic cancers during chemotherapy. Enteric nutritional supplements containing omega-3 fatty acids (Racol^®^) was administered to aforementioned patients with cancers during chemotherapy. The skeletal muscle mass and blood test data were obtained pre-administration and 28 and 56 days after. Patients with pancreatic cancer were administered the digestive enzyme supplement pancrelipase (LipaCreon^®^) 28 days after the start of Racol^®^ administration. The number of chemotherapies skipped due to neutropenia was recorded for 2 months before and after enteral nutrient initiation. In all 39 patients, the skeletal muscle mass increased on day 56 versus baseline (median 17.3 kg vs. 14.8 kg, *p* < 0.01), number of chemotherapies skipped decreased (mean: 0.65 times/month vs. 1.3 times/month, *p* = 0.03), and retinol-binding protein (mean: 2.56 mg/dL vs. 2.42 mg/dL, *p* = 0.05) increased. Patients with pancreatic cancer showed increased blood eicosapentaenoic acid concentration on day 56 versus baseline (median: 48.1 μg/mL vs. 37.0 μg/mL, *p* = 0.04) and increased skeletal muscle mass (median 16.8 kg vs. 14.4 kg, *p* = 0.006). Baseline median neutrophil count increased significantly from 2200/μL at baseline to 2500/μL (*p* = 0.04). Patients with biliary tract cancer during chemotherapy also exhibited increased skeletal muscle mass following omega-3 supplementation (median 17.3 kg vs. 15.8 kg, *p* = 0.01). In patients undergoing chemotherapy for unresectable or post-recurrence pancreatic and biliary tract cancers, high-omega-3 fatty acid nutrition therapy use improved skeletal muscle maintenance and chemotherapy dosing intensity.

## Introduction

Cancer cachexia is a complex metabolic disorder characterized by progressive loss of skeletal muscle that is difficult to improve with conventional nutritional therapy. Its pathophysiologic mechanism involves protein catabolism and energy imbalance caused by varying interlockings of anorexia and metabolic disorders [1]. Cachexia occurs in approximately 80% of patients with advanced-stage cancer, with a high incidence in those with pancreatic, stomach, and lung cancer [2]. Patients with advanced-stage cancer are often forced to receive chemotherapy. Continued chemotherapy has a significant impact on the patient’s prognosis [3]. The loss of body weight and skeletal muscle mass increases the prevalence of adverse events during chemotherapy and contributes to a worsening prognosis in cancer patients [4–7]. Malnutrition is caused by changes in the neurohormonal mechanism that controls food intake, leading to metabolic changes that contribute to a negative energy balance. It affects protein turnover and inflammation, thereby promoting muscle wasting, as observed in patients with cachexia [8]. In cachexia patients, both the tumor and the host produce several cytokines that are thought to be molecular mediators of the metabolic modification of cachexia [9, 10].

Therefore, nutritional interventions using substances with both nutritional and anti-inflammatory properties have been proposed to improve energy balance and reduce inflammatory conditions. From this perspective and in light of their anti-inflammatory properties, fish oil and its primary components, omega-3 fatty acids, have been proposed as treatment options for cachexia. Nutritional interventions with omega-3 fatty acids have been shown to help maintain weight and improve chemotherapy completion rates [11–13].Moreover, omega-3 fatty acids have an anti-inflammatory effect [14]. Recent studies have reported that when omega-3 fatty acid-containing enteric nutritional supplements were administered in patients with pancreatic and biliary tract cancer during the course of chemotherapy, their skeletal muscle mass increased, suggesting that this supplement can improve cancer cachexia [15]. This study added new patient data to the previous report, reanalyzing these data and examining the new impact of nutritional supplementation on the rate of continuing chemotherapy.

According to a recent study, the dose intensity of chemotherapy affects the prognosis of cancer patients, including those with pancreatic and biliary tract cancer [16]. A notable point of consideration is the relationship between omega-3 fatty acids and the number of chemotherapy sessions skipped, which has not been reported so far. This is especially crucial among patients with pancreatic and biliary tract cancer who often skip chemotherapy due to the occurrence of hematological toxicity and cholangiocarcinoma. As a result, chemotherapy is rendered less effective, consequently shortening the prognosis in several cases [16]. In the present study, in addition to the analysis on the effect of omega-3 fatty acids on cancer cachexia, the need for maintaining the dose intensity of anticancer drugs, which directly affects the patient’s prognosis, was also evaluated.

## Methods

### Patients and procedures

The study participants were patients with unresectable pancreatic and bile duct cancer who underwent chemotherapy between November 2014 and December 2019. Each patient was provided with 2–4 packs (200 kcal/300 mg of omega-3 fatty acid per pack) of an omega-3 fatty acid enteral nutrient (Racol^®^; Otsuka Pharmaceutical Factory, Tokyo, Japan) per day. The study participants (1) with a performance status (PS) of 3 and 4, (2) with difficulties in oral ingestion, (3) with active infectious disease, (4) with a history of hypersensitivity to administered drugs (supplements), (5) with uncontrolled diabetes mellitus, (6) with double cancer other than that in the biliopancreatic region, (7) who were noncompliant (who took two or fewer drugs per day), (8) with difficulties in continuing chemotherapy during the study period, (9) who were under warfarin treatment, and (10) with a platelet count of < 100,000/ μL were excluded. The outcome measures were assessed before treatment and 28 and 56 days after the initiation of treatment. Each patient was asked to consume approximately the same number of calories every day (the exact calorie intake was not calculated). After undergoing pancreatic function diagnostic tests (to assess the para-aminobenzoic acid excretion rate and pancreatic exocrine function), patients with pancreatic cancer were additionally administered with the pancreatic digestive enzyme supplement LipaCreon^®^ (150 mg, 12 times/day; Abbott Japan, Tokyo, Japan) from the 28th day of treatment onward. This study was approved by the Ethics Committee of the Jikei University School of Medicine [review number: 26-070(7575)]. All procedures were performed in accordance with the ethical standards of the responsible committee on human experimentation and with the 1975 Declaration of Helsinki, as revised in 2008. Informed consent was obtained from all patients prior to the study inclusion.

### Measures

The primary outcome measures were skeletal muscle mass and the number of chemotherapy sessions skipped. As primary end points, skeletal muscle mass was measured through a bioelectrical impedance analysis using the body composition meter DF860K^®^ (Yamato Scale CO., LTD., Hyogo, Japan). The rate of chemotherapy continuation was evaluated based on the number of chemotherapy sessions skipped due to neutropenia 2 months prior to and after study initiation. Body weight and blood sampling data (retinol-binding protein [RBP] level as a marker of nutritional status, neutrophil count, serum EPA level, carcinoembryonic antigen level, and glycated hemoglobin [HbA1c]) were collected as secondary end points.

### Statistical analyses

Data were expressed as mean ± standard error (SEM). All evaluation items were compared using the Wilcoxon signed-rank test. A *p* value of < 0.05 was considered statistically significant. These analyses were conducted using IBM® SPSS version 20.0 (IBM Japan, Tokyo, Japan).

## Results

**Fig. 1:**
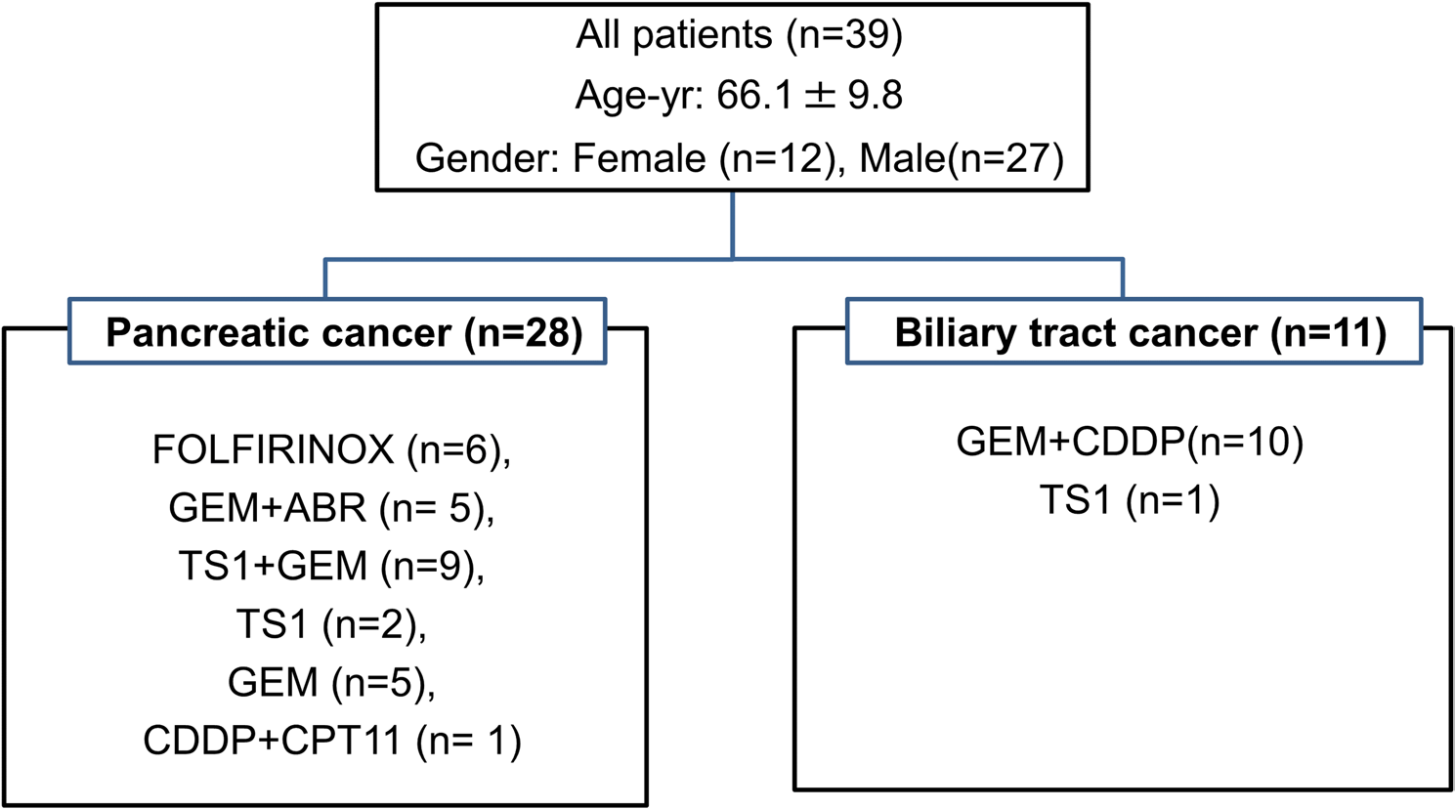
Patients’ background and chemotherapy regimen. Leucovorin and fluorouracil plus irinotecan and oxaliplatin (FOLFIRINOX), gemcitabine (GEM), abraxane (Abr), irinotecan (CPT 11), and cisplatin (CDDP)

**Fig. 2:**
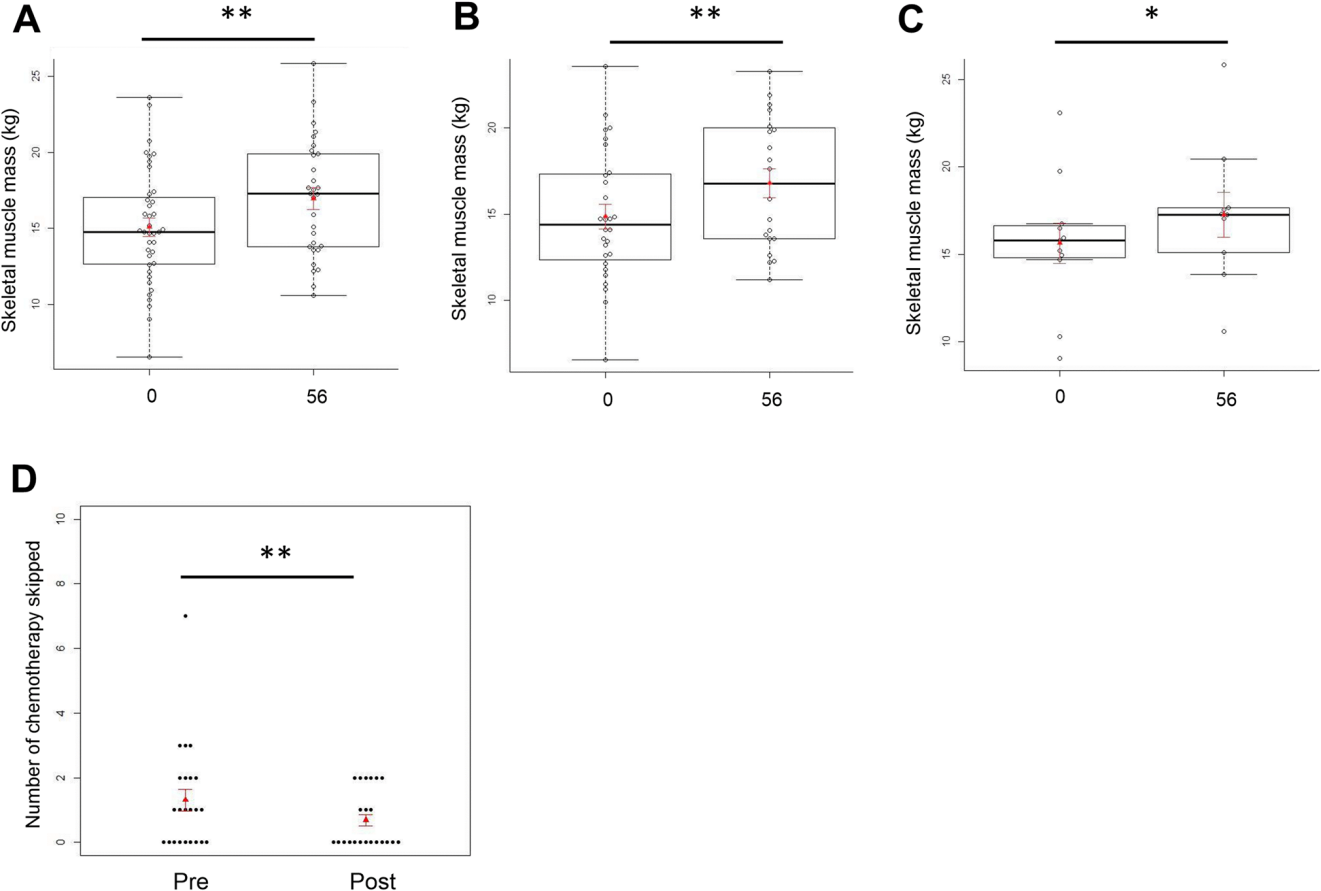
Primary outcome measures of drug response. Accessing the skeletal muscle mass, all patients (**A**); patients with pancreatic cancer (**B**); patients with biliary tract cancer (**C**); and the number of chemotherapy sessions skipped in all patients (**D**). Data are expressed as mean ± SEM (red lines). ** *p* < 0.01. * *p* < 0.05 (vs. day 0) and Wilcoxon rank-sum test. The numbers (0, 56) on the horizontal axis denote the days after the start of treatment

This study included 39 patients who received chemotherapy—11 with biliary tract cancer and 28 with pancreatic cancer, including one with neuroendocrine cancer. The participants consisted of 27 men and 12 women with an average age of 66.1 years. The chemotherapy regimens used for the treatment of pancreatic cancer were as follows: leucovorin and fluorouracil plus irinotecan and oxaliplatin (FOLFIRINOX) in six patients, gemcitabine (GEM) + abraxane (Abr) in five patients, TS1 + GEM in nine patients, TS1 in two patients, GEM in five patients, and cisplatin (CDDP) + irinotecan (CPT1) in one patient. The chemotherapy regimens used for the treatment of biliary tract cancer were as follows: GEM + CDDP in 10 patients and TS1 in 1 patient (Fig. 1). All patients were reported to meet the sarcopenia diagnosis criteria based on their skeletal muscle mass, which was measured prior to the start of the study. Patients with pancreatic cancer had decreased exocrine pancreatic function prior the start of nutrition therapy (mean ± standard deviation; 40 ± 18% in pancreatic function diagnostic test). The average Racol^®^ intake was 2.32 packs. On day 56 of the nutrient supplementation, all patients exhibited increased body weight (median: 62.4 kg vs. 58.9 kg, *p* = 0.03) (Table 1), increased skeletal muscle mass (median: 17.3 kg vs. 14.8 kg, *p* < 0.01) (Fig. 2A), decreased number of chemotherapy sessions skipped (mean: 0.65 times/month vs. 1.3 times/month, *p* = 0.03) (Fig. 2D), and increased RBP level (mean: 2.56 mg/dL vs. 2.42 mg/dL, *p* = 0.05) (Table 1) compared with those before administration. The HbA1c levels were almost unchanged, and the glucose tolerance abnormalities did not worsen after administering nutritional supplements (day 56 vs. pre-administration: median 6.5% vs. 6.4%, no significant difference, *p* = 0.87) (Table 1). In patients with pancreatic cancer, on day 56, the blood EPA concentrations (median: 48.1 μg/mL vs. 37.0 μg/mL, *p* = 0.04) (Table 1), skeletal muscle mass (median: 16.8 kg vs. 14.4 kg, p = 0.006) (Fig. 2B), and neutrophil counts significantly increased (median: 2200/μL vs 2550/μL, *p* = 0.04), compared with those before the administration of supplements (Table 1). The administration of LipaCreon®, a pancreatic digestive enzyme supplement, for 4 weeks did not show any additional effect in increasing the skeletal muscle mass or improving the nutritional status of patients taking omega-3 fatty acids (Table 1, Fig. 2A, B). Patients with biliary tract cancer showed an increase in skeletal muscle mass on day 56 compared with that before administration (median: 17.3 kg vs. 15.8 kg, *p* = 0.01) (Fig. 2C).

**Table 1:**
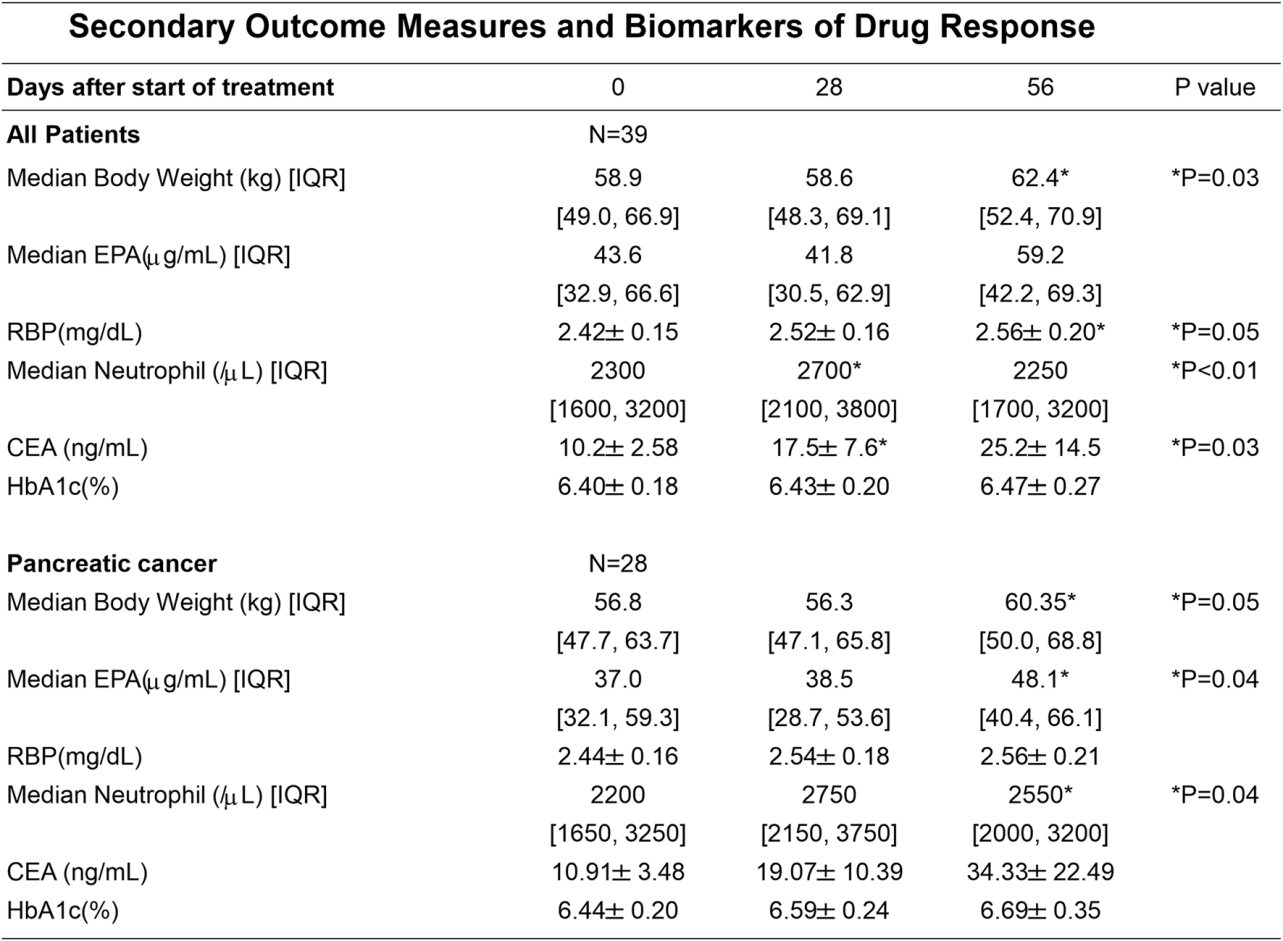
Data are presented as mean ± SEM. *A *p* value of <0.05 (vs. day 0) was considered significant. Pre, before intervention; post, after intervention

## Discussion

Omega-3 fatty acids increase the skeletal muscle mass and improve the severity of sarcopenia [14]. When omega-3 fatty acid was administered to patients with cancer of the lungs [17, 18], stomach, colon, rectum [19], pancreas [21–23], head and neck [20], and biliary tract [12], their weight, blood test data (nutrition index and neutrophilia), and quality of life (QOL) score improved, suggesting that omega-3 fatty acid has a therapeutic effect on cancer cachexia. However, these studies have inconsistencies. First, the severity of cachexia in patients with cancer was evaluated based on the body weight. Patients with advanced-stage cancer are at risk of experiencing fluid retention in the third spaces, such as edema and pleural effusion. The skeletal muscle mass is an appropriate indicator of cancer cachexia. Since the skeletal muscle mass is considered to correlate with activities of daily living and PS in cancer patients, it is useful for evaluating the sarcopenia-improving effect of omega-3 fatty acid [24–26]. Second, the causal relationship between nutritional status, blood sampling items, and omega-3 fatty acid levels (e.g., blood EPA levels) has not been evaluated. Third, although the incidence of chemotherapy-related adverse events has been investigated [11,17,26], the need for continuing the dose intensity of anticancer drugs, which directly affects the prognosis of patients, has not been evaluated. In order to resolve the aforementioned issues, a more useful evaluation index was utilized in patients with pancreatic and biliary tract cancer who received chemotherapy. Therefore, we investigated whether nutrition therapy using omega-3 fatty acids was effective in improving sarcopenia. This nutritional therapy increased the skeletal muscle mass and improved the nutritional status, suggesting that it contributed to the improvement of patients’ QOL. The administration of omega-3 fatty acids in patients with pancreatic cancer prevented the occurrence of weight loss [22, 23].

Cancer cachexia is caused by inflammatory cytokines produced by cancer cells. This promotes skeletal muscle catabolism and leads to a decrease in skeletal muscle mass, which is a criterion for diagnosing sarcopenia. Omega-3 fatty acid has an inhibitory effect on inflammatory cytokines [8], and its effectiveness against cachexia in patients with pancreatic cancer has already been reported [27]. Our study results also suggest that skeletal muscle loss was suppressed by the anti-inflammatory effect of omega-3 fatty acid. Generally, the skeletal muscle mass of a healthy person accounts for 30%–35% of the total body weight [28]. In our study group with prominent sarcopenia, the administration of omega-3 fatty acid led to the recovery of skeletal muscle mass to the pre-sarcopenia levels.

This study investigated whether the combined use of a pancreatic digestive enzyme replacement agent and an enteric nutritional supplement would improve the absorption of omega-3 fatty acids and exert a stronger drug effect in patients with pancreatic cancer with exocrine pancreatic insufficiency. However, the administration of a pancreatic digestive enzyme replacement agent for 4 weeks did not show any additional effect in increasing the skeletal muscle mass or improving the nutritional status of patients taking omega-3 fatty acids. Hence, future studies should examine the extent of the pancreatic digestive enzyme replacement dose and administration period.

In addition, in a combined analysis of patients with pancreatic and biliary tract cancer, nutritional therapy with omega-3 fatty acids significantly reduced the rate of skipping chemotherapy sessions (*p* = 0.03), indicating that the dose intensity was maintained; this may contribute to the improvement in the prognosis of patients. In each subgroup analysis of pancreatic cancer and biliary tract cancer, the number of chemotherapy sessions skipped tended to decrease in each group, but no significant difference was observed (*p* = 0.19 and *p* = 0.089, respectively). A detailed study on the number of chemotherapy sessions skipped in each cancer patient may require the use of a large sample size.

This study has some limitations. The enteric nutrients used in this study have the highest content of omega-3 fatty acids among those currently used in Japan; other nutrients, such as carbohydrates, proteins, and vitamins, are also included. In Japan, a single administration of omega-3 fatty acid is not covered by the insurance unless the patient has a certain disease such as arteriosclerosis obliterans. It is possible that nutrients other than omega-3 fatty acids were also involved in improving sarcopenia because of their abundant content. In recent years, a number of studies on cell systems, pre-clinical mammalian models, and humans have been conducted and have demonstrated the positive influence of omega-3 fatty acid intake on the skeletal muscle [29–32]. Therefore, omega-3 fatty acids promote protein synthesis in skeletal muscle and are involved in maintaining the skeletal muscle mass. Since the levels of omega-3 fatty acids in the blood of pancreatic cancer patients was significantly increased in this study, the content of omega-3 fatty acid in the formulation used might contribute to the increase in skeletal muscle mass. Enteronutrients only serve as supplements to the normal diet, and our study suggests that an additional intake of omega-3 fatty acids of 600 mg per day is sufficient.

In this study, we investigated high-omega-3 fatty acid nutrition therapy in patients with unresectable or recurrent biliary or pancreatic cancer during chemotherapy, which improved skeletal muscle maintenance and chemotherapy dosing intensity. Due to the similar nature of both diseases, pancreaticoduodenectomy has often been selected as the initial surgery in both cases [33]. In cases of recurrence, surgery is not indicated, and chemotherapy is preferred. However, despite the administration of chemotherapy in these cases, the average overall survival is only less than 1 year, and the PS remarkably decreases [33]. Another common feature is the high incidence of local recurrences and liver metastases in patients with disease recurrence [34, 35]. Furthermore, GEM is commonly used as the chemotherapy regimen, which is mainly combined with TS1 [16,35]. Strictly speaking, a study must be conducted using a chemotherapy regimen, but this is difficult to implement in those with pancreatic and biliary tract cancers since the regimen is often changed or discontinued due to the decrease in PS, refractory treatment effect, and onset of jaundice and cholangitis. Additionally, all patients, including those with pancreatic and biliary tract cancer, were investigated as sarcopenia patients; in the subgroup analysis, patients with pancreatic and biliary tract cancer were evaluated. Therefore, improvement was recognized.

## Conclusion

Omega-3 fatty acid-rich nutritional therapy for patients undergoing chemotherapy for unresectable or post-recurrence pancreatic and biliary tract cancers increased the skeletal muscle mass and improved the chemotherapy dosing intensity without major adverse events.

## Data Availability

The datasets generated or analyzed in the present study are included in this publication and are available on reasonable request.
